# Social Capital as a Determinant of Pregnant Mother’s Place of Delivery: Experience from Kongwa District in Central Tanzania

**DOI:** 10.1371/journal.pone.0138887

**Published:** 2015-10-01

**Authors:** Innocent Antony Semali, Germana Henry Leyna, Elia John Mmbaga, Anna Tengia-Kessy

**Affiliations:** 1 Department of Epidemiology and Biostatistics, School of public Health and Social sciences, Dar es Salaam, Tanzania; 2 Department of Community Health, School of public Health and Social sciences, Dar es Salaam, Tanzania; Örebro University, SWEDEN

## Abstract

**Introduction:**

Maternal ill health contributes highly to the global burden of diseases in countries South of Sahara including Tanzania. Ensuring that all deliveries take place in health facilities and hence attended by skilled health personnel is one of the strategies advocated by global and national policies, including the Millennium Development Goals (MDGs). However, the number of women delivered by skilled health personnel has remained low in sub Saharan Africa despite of a number of interventions. We sought to determine the role of social capital in facilitating health facility delivery.

**Methods:**

We randomly selected 744 households with children aged less than five years from two randomly selected wards in a rural area in Tanzania. Mothers were enquired about place of delivery of the last child. Social capital was assessed using a modified questionnaire with both structural and cognitive aspects of social capital, administered in face-to-face interviews. Principal Component Analysis (PCA) was used to develop asocial capital index measure. Uni-variate and multivariable regression models were run using STATA 12.

**Results:**

Majority (85.9%) of the mothers reported to have delivered in a health facility during their last birth. Compared to the lowest social capital quintile, delivering in a health facility increased significantly with increase in social capital level: low (Adjusted Odds Ratio (AOR) = 2.9; Confidence Interval (CI): 1.4–6.1, p = 0.004); moderate (AOR = 5.5, CI: 2.3–13.3, p-value<0.001); high (AOR = 4.7; CI: 1.9–11.6, p-value<0.001) and highest (AOR = 5.6, CI: 2.4–13.4, p-value<0.001) and χ^2^-test for the trend was significant (χ^2^ = 17.21, p<0.001).

**Conclusion:**

Overall, social capital seems to play an important role in enhancing health facility delivery that may lead to improved maternal and child health. Concerted efforts should focus on promoting and supporting effective social capital and in particular cognitive social capital.

## Introduction

Global burden of diseases is estimated at 2.5 billion years of life lost due to diseases and injuries, of which maternal causes contribute0.9%[1]. Approximately 800 women die every day because of maternal causes and 99% of them occur in developing countries[2]. Comparatively, a higher proportion of burden of maternal causes is found in Sub-Saharan Africa, despite the region being home to only 10% of the global population. Since 1990, various global efforts have reduced maternal mortality by 47% (from 400 to 210 per 100,000 live births) by the year 2010[3].But, maternal mortality remains high (500 per 100,000 live births) in Sub-Saharan Africa. For example the maternal mortality in Tanzania is estimated to have declined from 900 per 100,000 to 700 to 454 from 1990, 2000 and 2010, respectively [4].

Major causes of maternal mortality in developing countries are preventable and some could be easily addressed if a skilled attendant is present during each birth. The rates of deliveries attended by a skilled health worker have been reported to be low in developing countries. A recent study in Bangladesh revealed that only 48.9% of deliveries were assisted by a skilled attendant [5] while in Tanzania, only 51%of all births are attended by skilled attendants[4]. Research suggests that high maternal education; high wealth level and antenatal clinic attendance significantly influence the likelihood of skilled attendance at birth[6].Other factors influencing place of delivery include the ages of both parents, gravidity, marital status, education and other demographic characteristics [7, 8].

Furthermore additional household characteristics that influence utilization of health services and place of giving birth include the number of people in the household and wealth [9]. Thus, households with fewer people and or children are more likely to make a decision for the woman to deliver in a health facility. Level of agricultural activities such as farm sizes and animals kept in a household have also been reported to influence a place where a pregnant mother would deliver. On the other hand, community level factors that influence place of delivery include distances to markets, water sources and health facility[8] and whether or not the facility offered comprehensive emergency obstetric care[10]. Thus, associations between individual, household and health facility factors with facility delivery are well established.

However, the role of community level contexts and especially social capital influencing reproductive health decisions such as health facility delivery and skilled birth attendance has received limited attention in Sub-Saharan Africa. Social capital is defined as “features of social organization, such as civic participation, norms of reciprocity, and trust in others, that facilitate cooperation for mutual benefit”[11]. The two main categories of social capital are structural social capital which includes membership, networks, organization support and collective action, and cognitive social capital which includes solidarity, trust, cooperation and conflict resolution[12]. Social capital is the ability to secure benefits through memberships in networks and other social structures[13]. The core networking in social capital is highly dependent on trust, reciprocity, expectations, and obligations. Benefits of social capital are through social control and support which include health, economic, security, educational, overall access to services and development. For example, the marked social economic variation across regions in Italy was not explained by the baseline economic status of the regions, but rather by the level and degree of civic organizations and hence social capital[14]. The north which had the most civic organizations also had the highest level of social economic development including health.

The relationship between income, economic wellbeing and social capital is explained by the position of the individual in the community relative to others, rather than absolute income[15]. It has also been observed that mortality level is related to the distribution of income and hence the equity existing in a particular area[16–18]. The pathway through which social economics affect health is believed to be through social capital. A strong correlation between social capital indicators and income inequality and overall mortality have been observed[19]. In addition, communities with strong social capital have been observed to have significantly better health outcomes as compared to those without[20]. It suggests that social capital has the ability to minimize the effects of social economic inequality on health. This is also supported by studies elsewhere which document that social capital has the potential to favorably influence exposure to health determinants, health status as well as utilization of health care and its outcomes [21–24].Though the interest on the role of social capital in developing countries is recent and on the rise, benefits differ between countries and between sub-units within a country[25]. Using a cross sectional study, we aimed to assess the influence of social capital combined on health facility delivery in rural Tanzania.

## Methodology

### Study design

We conducted a cross-sectional study in Kongwa,a rural district in Dodoma region in central Tanzania. The district is divided into three wards which are further subdivided into 16 villages, and each village is obliged to maintain a regularly updated register of all its members. The district public health care facilities include one hospital, three health centers and eight dispensaries. All these facilities provide maternal and child health services. In addition, there are scheduled outreach services for villages which are far from the health care facilities. These outreach services provide antenatal care and counsel women to deliver in health facilities.

### Power calculation and sample selection

Based on the rate of health facility delivery in Dodoma region of 45.9% (less than the national average which was 50.6%) [4], a sample size calculation was done to achieve 80% power to detect differences in place of delivery for different social capital quintiles, controlling for village as cluster. Two wards namely, Mlali with a population of 19,623 (10,318 females) and Ugogoni with a population of 17,048 (8,989 females) were randomly selected from the three wards of Kongwa District. All villages in the sampled wards were included in the study. A list of all households (people living together and eating from the same pot) in each village was obtained from the village office and households with a child aged less than five years were identified and listed. Systematic random sampling was then applied to select the required number of households from each village, proportional to the number of eligible households in that particular village (sampling proportional to size). At the household level, the mother of the child was interviewed and in those households where there was more than one child, the mother with the youngest child was selected for interview. A total of 744 mothers with children aged less than five years were interviewed, and there were no refusals.

### Data collection tools

A structured questionnaire was developed to collect information on child socio-demographic characteristics and utilization of health services, including whether or not the delivery of the youngest child took place in a health care facility. Other questions focused on household characteristics. Furthermore, the validated World Bank's social capital assessment tool on household social capital survey was used to collect household data on structural and cognitive aspects of social capital at both individual and collective levels[26]. Some of the questions on socio-demographic and household characteristics were adopted from the demographic and health survey questionnaire, which is well validated and widely used.

The tools were originally in English, it was translated to Kiswahili the media of interview. The Kiswahili version was back translated to English by different people until the two versions were consistent. The final Kiswahili version was then used to train the research assistants and later a final draft was produced. However, we pre-tested the questionnaire in a similar population in the district and made appropriate adjustments to ensure validity.

### Data collection process

Prior to the survey, the research team met with the respective village leaders, informed them of the objectives of the study and requested them to inform the expected participants of the team visits in their households. The survey team, which consisted of trained research assistants, went house to house with the help of the village leaders, administering the questionnaire in face-to-face interviews.

### Study variables

The main independent variable was social capital. Questions on social capital comprised of sub-sections on both structural and cognitive social capital. Each item in the questionnaire was coded “1” for a “yes” response and indicated positive social capital and “0” if it was “no”, indicative of negative social capital. Principal Component Analysis (PCA), a statistical approach used to reduce a large set of variables to a small manageable set was used to identify main components of the social capital tool. According to this approach, the main components identified accounted for most of the variability in the data as also obtained when using the Kaiser criterion (Eigen value >1) and Scree plots. The first component was then adapted as a social capital index, which was used to create social capital quintiles. The least was the most disadvantaged level and the fifth quintile reflected best level of social capital.

The other independent variable was wealth index. This was developed using asset ownership which included radio, bicycle, hand phone, cooker, television, refrigerator, agricultural equipment, quality of housing and source of energy. Assets data was also subjected to PCA using similar approach as described for the social capital variable. The component with the highest loading was adopted as wealth index. Wealth quintiles were then created from the wealth index, with the first quintile being the least and labeled the poorest while the fifth was labeled the highest.

Additional independent variables included education of both mother and head of the household. It was categorized into less than primary school coded “0” and above coded as “1”, marital status coded “1” if married and “0” otherwise, age below 30 years coded as “1” and “0” otherwise. Others were household size coded as “1” if less than five and “0” otherwise, number of children coded “1” less than five, distance to health facility coded “1” if less than 5 kilometers and walking time to nearest water source coded as “1” if less than 30 minutes and “0” otherwise.

The dependent variable was place of birth of the last child and thus all health facility deliveries were categorized as deliveries under skilled attendance and coded “1” or otherwise coded “0”. All mothers had antenatal and child monitoring cards and these were used to verify place of birth.

### Data management and analysis

The data was checked for completeness and consistency followed by generation of appropriate frequencies and descriptive statistics using STATA version 12. Given that dependent variable was dichotomous a command for multilevel exposure analysis (tabodds) was used in the bivariate and multivariate analyses producing similar results to logistic regressions. “Tabodds”, a STATA command tabulates unadjusted and adjusted odds ratios (OR), for each level of the exposure variable against a reference level which by default was the lowest level of the social capital quintile variable[27]. In addition, it performed an approximate chi-square test of homogeneity of the ORs and a trend test for a linear trend of the log odds against the levels of the explanatory variable, which also serves as a test for dose–response if the exposure variable is in ordered (hierarchical) levels. In both functions, the chi square statistics was reported. Thus as logistic regression it determined the unadjusted (crude) Odds Ratio (COR) for the association of dependent and independent variables, followed with adjusted Odds Ratio (AOR) which adjusted for potential confounding effect of the social demographic characteristics. Significance level was set at a p-value ≤0.05.

### Ethical consideration

The study was reviewed and ethically cleared by the Muhimbili University of Health and Allied Sciences Ethical Review Committee. Permission to conduct the study was granted by regional, district, ward and village authorities. All participants gave written informed consent before participation and research information was kept confidential. Furthermore, no names were recorded on the survey tools.

## Results

The study recruited 744respondents; mostly (67.9%) young mothers aged less than 30 years (Table 1). Majority (78.6%) of the mothers reported to be married at the time of the survey and slightly over half (57.1%) of them had attained primary or higher education. A high proportion (85.9%) of births took place in a health care facility. Majority of the mothers (81.6%) had to walk five kilometers or more to reach the nearest health care facility whereas three-quarters (74.6%) of these mothers reported to walk for 30 minutes or more to reach a water source. Most of the respondents (57.4%) came from households with large family size (more than five people) and owned farms (80.9%) with half (51.3%) of them owning less than 4-acre farms. About a fifth (21.3%) of the respondents was from households classified in the lowest wealth quintile.

**Table 1:** Household and socio-demographic characteristics of respondents (N = 744).

Characteristic	Frequency	Percent
**Household characteristics**		
Head’s age over 30 years	330	55.6
Head education level: 7 years and more	501	67.3
Household size: 5+	427	57.4
Number of children: <5	597	80.2
Household owns a farm	602	80.9
Number of acres: <4	382	51.3
Household owns livestock	372	50.0
**Wealth quintile**		
Lowest	152	21.3
Low	136	19.2
Moderate	146	20.1
High	138	19.3
Highest	142	19.9
Distance to nearest facility: ≤5 Kms	607	81.6
Walking time to water source: ≥30 minutes	555	74.6
**Maternal characteristics**		
Age of the mother <30 years	505	67.9
Marital status: Married	577	78.6
Education level: ≥7 years	425	57.1
Health facility delivery, yes	639	85.9

Table 2 presents the bivariate and multivariable regression analysis of social capital and selected household and maternal predictors of health facility delivery of the most recent birth. Social capital has a positive significant influence on the probability of health facility delivery. Compared to the lowest social capital quintile, the likelihood of health facility delivery increased with increasing social capital quintile, AOR = 2.9 (CI: 1.4–6.1), 5.5 (2.3–13.3), 4.7 (1.9–11.6) and 5.6 (2.4–13.4) respectively for the low, moderate, high and highest social capital quintiles. Furthermore the chi square test for linear trend of social capital quintiles and facility delivery was statistically significant (χ^2^-test trend = 17.21, p<0.001).

**Table 2:** Bivariate and multivariable logistic regression of social capital and select household and maternal predictors of health facility delivery.

Variable	Category	COR* (95% CI**)	AOR^§^ (95% CI)	P-value
Head’s age (years)	≥ 35	1.0	1.0	
	<35	1.4 (0.9–2.2)	1.7 (1.0–2.8)	**0.051**
Head education level, years	<7	1.0	1.0	
	≥ 7	2.1 (1.4–3.3)	1.9 (1.2–3.2)	**0.004**
Household size	< 5	1	1	
	≥ 5	1.4 (0.9–2.1)	0.99 (0.6–1.6)	0.961
Number of children	< 4	1	1.0	
	≥ 4	1.8 (1.1–2.9)	1.68 (1.0–2.9)	**0.057**
Wealth quintiles	Lowest	1.0	1.0	
	Low	1.4 (0.8–2.5)	1.7 (0.8–3.3)	0.145
	Moderate	1.6 (0.9–2.9)	1.6 (0.8–2.9)	0.170
	High	2.4 (1.2–4.7)	2.1 (1.0–4.2)	**0.036**
	Highest	2.9 (1.4–5.9)	3.3 (1.0–4.2)	**0.002**
Distance to nearest facility, Km	≥ 5	1		
	< 5	3.6 (1.6–7.9)	3.41 (1.3–8.8)	**0.007**
Time to water source, minutes	< 30	1		
	≥ 30	1.1 (0.7–1.7)	0.93 (0.5–1.6)	0.802
Age of the mother, years	< 30	1	1.0	
	≥ 30	1.4 (0.9–2.2)	1.3 (1.0–3.9)	**0.057**
Married	No	1.0	1.0	
	Yes	1.0 (0.4–1.1)	0.66 (0.4–1.2)	0.149
Education level mothers, years	< 7	1	1.0	
	≥ 7	1.9 (1.3–3.0)	1.67 (1.1–2.7)	**0.029**
Social capital quintiles^**t**^	Lowest	1.0	1.0	
	Low	2.3 (1.4–4.8)	2.9 (1.4–6.1)	**0.004**
	Moderate	3.4 (1.7–6.6)	5.5 (2.3–13.3)	**<0.001**
	High	3.4 (1.7–6.6)	4.7 (1.9–11.6)	**<0.001**
	Highest	3.3 (1.7–6.5)	5.6 (2.4–13.4)	**<0.001**

* COR—Crude Odds Ratio

* * CI—Confidence Interval

^§^ AOR—Adjusted Odds Ratio, adjusted for social demographic characteristics.

^**t**^χ^2^-test trend = 17.21, p<0.001.

Household characteristics observed to positively influence health facility delivery included: being in highest two wealth quintiles (high wealth quintile (AOR = 2.5; CI:1.0–4.2; p = 0.036) and highest wealth quintile (AOR = 3.3; CI:1.0–4.2; p = 0.002), living close to a health facility (≥5 Km) (AOR = 3.4; CI:1.3–8.8; p = 0.007) and the head of household having seven years or more of education (AOR = 1.9; CI:1.2–3.2; p = 0.004).

Maternal education of 7 or more years increased the likelihood of health facility delivery by 90% (AOR = 1.9, 95%CI: 1.3–3.0; p = 0.029). Furthermore, maternal age had a borderline significance influence on the likelihood of a health facility delivery (AOR = 1.3; CI: 1.0–3.9; p = 0.056). Likewise, the age of the head of the household (p = 0.051) and having four or more children in the house (p = 0.057) had a borderline association with delivery in a health facility.

## Discussion

We aimed to determine the influence of social capital on health facility delivery in rural Tanzania. Majority of the mothers reported a health facility delivery for the last child in the household. High level of social capital was a significant positive predictor of a health facility delivery.

Improving the rates of health facility delivery in sub Saharan Africa is one of the many interventions aimed at reducing maternal and infant mortality rates in these countries. We observed a high reported health facility delivery than the recently reported national average of 50.6%[4]. Although our findings are higher (85.9%) than the reported national average, regional variations reveal regions with higher than average facility delivery such as Kilimanjaro (86.1%), Ruvuma (83.0%) and Dar es Salaam (91.0%) regions[4]. However, most studies from other African settings have reported lower proportions of births taking place in a health facility[28]. Our results may reflect achievements in a district doing well over and above the national average as a result of ongoing district-specific interventions like the outreach services. A similar trend in increase in health facility delivery is observed at the global level where countries report different levels of success towards achieving the MDGs on maternal and child health[29–31]

Education of the mother and of the head of the household was significantly associated with health facility delivery. Similarly, a study elsewhere on determinants of facility delivery revealed education of the mother and of the partner; distance to health facility as well as attendance to antenatal care to be significantly associated for the likelihood of delivering at a health care facility[8]. Better education could facilitate compliance to maternal and child instructions while it can also enhance access to resources that would facilitate the utilization of delivery services. Thus increasing education attainment and reducing distance to health facility could play an important role in the utilization of maternal care services. Our study revealed an association between the age of both mother and the head of the household with delivery at a health facility. These findings are supported by a study elsewhere which reported an increased likelihood of facility delivery if the mother was younger[32].

Social capital (or the relationships, attitudes and values that govern interactions among people) has been described to have various positive outcomes ranging from economic and social development and more recently to health outcomes. Both forms of social capital i.e. structural and cognitive social capitals have been shown to inversely influence health [33–35] although current evidence suggests that the differences in health are better predicted by cognitive social capital[36]. Some studies have argued for multi-level analysis to parse out the cross-level interaction between individual (cognitive) and community level (structural) social capital [37]. We observed a 3–6 fold significant increase in the likelihood of reporting a health facility delivery with increase in the level of social capital after accounting for socio-economic and demographic characteristics of the head of household and the mother. Our findings follow trends that have been reported from both developed countries and other low and middle income countries [38–41]. The mechanism to which social capital influences health is not clearly known. It has been suggested that social capital may facilitate the spread of knowledge on health and health promotion, promote healthy behavior by providing access to health services and amenities or by providing support and mutual trust among members in a social network [12, 42]. The recent increase in the number of women groups for micro credit as well as social purposes in Tanzania may contribute to the above mechanism as personal bonds characterized by trust and shared values are common in these groups.

Our study was carried out in a rural setting using an adapted social capital tool that assessed both structural and cognitive forms of social capital. We translated the tools which was originally in English to Kiswahili the media of interview. The Kiswahili version was back translated to English by different people until the two versions were consistent which also assured that respondents had uniform understanding of the tool. However, our tools are prone to reporting bias and may have led to overestimation of the rates of health facility delivery and/or social capital. Due to the lack of knowledge on the hypothesis to be tested (by the respondents) we believe the bias was non-differential and thus have not attenuated the true effect of social capital on health facility delivery.

Social capital is increasingly being shown as an avenue that can be used to promote health and health outcomes in low- and middle-income countries. Governments and policy makers should facilitate and use this channel to fasten and sustain progress made towards achieving the MDG on maternal health. In addition, more efforts are needed to parse out the pathways through which social capital influences health to further inform the ongoing development strategies.

Findings from this study might have been limited by the fact that we studied mothers who survived the birth process and hence introduced a bias which might have overestimated the rate of facility deliveries. However the study employed questionnaires validated and used in other surveys including Tanzania DHS, thus the type of error and magnitude could be similar across such studies. Consequently, our results are validly comparable to those obtained elsewhere and across time, despite the inherent error.
